# Inaccurate diagnosis of diabetes type in youth: prevalence, characteristics, and implications

**DOI:** 10.1038/s41598-024-58927-6

**Published:** 2024-04-17

**Authors:** Mustafa Tosur, Xiaofan Huang, Audrey S. Inglis, Rebecca Schneider Aguirre, Maria J. Redondo

**Affiliations:** 1 The Division of Diabetes and Endocrinology, Department of Pediatrics, Baylor College of Medicine, Texas Children’s Hospital, Houston, TX 77030 USA; 2 Children’s Nutrition Research Center, Baylor College of Medicine, USDA/ARS, Houston, TX 77030 USA; 3 Institute for Clinical and Translational Research, Baylor College of Medicine, Houston, TX USA; 4 School of Health Professions, Baylor College of Medicine, Houston, TX USA; 5 Division of Diabetes, Endocrinology and Metabolism, Department of Medicine, Baylor College of Medicine, Houston, TX USA

**Keywords:** Pediatric diabetes, Type 1 diabetes, Type 2 diabetes, Diabetes, Misdiagnosis, Endocrinology, Endocrine system and metabolic diseases

## Abstract

Classifying diabetes at diagnosis is crucial for disease management but increasingly difficult due to overlaps in characteristics between the commonly encountered diabetes types. We evaluated the prevalence and characteristics of youth with diabetes type that was unknown at diagnosis or was revised over time. We studied 2073 youth with new-onset diabetes (median age [IQR] = 11.4 [6.2] years; 50% male; 75% White, 21% Black, 4% other race; overall, 37% Hispanic) and compared youth with unknown versus known diabetes type, per pediatric endocrinologist diagnosis. In a longitudinal subcohort of patients with data for ≥ 3 years post-diabetes diagnosis (n = 1019), we compared youth with steady versus reclassified diabetes type. In the entire cohort, after adjustment for confounders, diabetes type was unknown in 62 youth (3%), associated with older age, negative IA–2 autoantibody, lower C-peptide, and no diabetic ketoacidosis (all, p < 0.05). In the longitudinal subcohort, diabetes type was reclassified in 35 youth (3.4%); this was not statistically associated with any single characteristic. In sum, among racially/ethnically diverse youth with diabetes, 6.4% had inaccurate diabetes classification at diagnosis. Further research is warranted to improve accurate diagnosis of pediatric diabetes type.

## Introduction

Pediatric diabetes is one of the most common chronic conditions in childhood. Type 1 diabetes (T1D) and type 2 diabetes (T2D) account for most pediatric diabetes cases, and their incidences increased significantly over the past decades^1^. T1D is characterized by development of absolute insulin deficiency, typically of autoimmune origin, requiring lifelong insulin treatment while T2D is marked by relative insulin deficiency with insulin resistance and, at least initially, may respond to lifestyle or non-insulin agents^2,3^. However, overlaps in biological and clinical characteristics between different diabetes types pose significant challenges for clinicians^3^. Adding to the confusion, monogenic forms of diabetes, including neonatal and maturity-onset diabetes of the young (MODY), are relatively rare causes of pediatric-onset diabetes^4^ that may resemble T1D or T2D but require genetic testing for diagnosis. Atypical forms of diabetes or atypical presentations of common forms of diabetes are more frequent in non-European individuals^5–9^.

As each different diabetes type may require different treatment approaches, determination of diabetes type at diagnosis of diabetes guides clinical management decisions in both short- and long-term diabetes care^3^. It also shapes the type and depth of diabetes education and counseling that families receive during their clinical encounters. In addition, clinicians follow screening recommendations for commonly associated conditions (e.g., other autoimmune diseases) and micro-and macrovascular complications of diabetes based on the patient’s assigned diabetes type. Finally, diabetes risk in family members and approaches to prevention vary by diabetes type. Therefore, accurate determination of diabetes type at the onset of diabetes is key to providing optimal diabetes care.

Although there is heightened interest in the challenges surrounding diagnosis of diabetes type^5,10–18^, the prevalence of inaccurate diagnosis of pediatric diabetes types and the factors associated with inaccurate diagnosis are not fully understood. Therefore, we aimed to study inaccurate diagnosis of diabetes type in racially and ethnically diverse youth. We hypothesized that a sizeable percentage of children with diabetes have an undetermined type of diabetes at diagnosis of diabetes and/or their assigned diabetes types are reclassified over time. Better understanding the frequency and characteristics of children with inaccurate diagnosis of diabetes type at onset will facilitate targeted interventions to address this problem.

## Methods

### Participants

In this retrospective study, we included individuals with any type of diabetes who were between 6 months and 20 years old at the time of diabetes diagnosis, had at least one subsequent outpatient visit between 2 weeks and 6 months post-diagnosis, and were seen per standard clinical protocol (which includes measurement of islet autoantibodies) at a Texas Children’s Hospital (TCH) Diabetes and Endocrinology Clinic between January 1, 2015, and February 1, 2022 (n = 2073, entire cohort). In addition, we studied a subcohort of patients who had at least an additional outpatient visit between 3 and 4 years post-diagnosis (n = 1019, longitudinal subcohort). The study was approved by the Baylor College of Medicine Institutional Review Board (IRB) (H-47418), and the requirement for informed consent was waived. All research was performed according to relevant guidelines and regulations.

### Procedures

With the assistance of a TCH electronic medical record (EMR) (i.e., Epic) data specialist, we generated a subject list meeting above inclusion and exclusion criteria, and extracted the following variables from the office visits that occurred after the diagnosis of diabetes and 3–4 years post-diagnosis: age, sex, race, ethnicity, body mass index (BMI) percentile, C-peptide, glucose, hemoglobin A1c (HbA1c), presence of diabetic ketoacidosis (DKA), and the results for islet autoantibodies (autoantibodies to Glutamic Acid Decarboxylase-65 [GADA], Islet Antigen 2 [IA–2A], Anti-insulin [IAA], and Zinc Transporter 8 [ZnT8A]). The race and ethnicity categorizations were based on self-report per documentation in the electronic medical record. We used the following racial and ethnic categories: (1) Races: White, African American, and Other races; and (2) Ethnicities: Hispanic and non-Hispanic. Other races include Asian, American Indian and Alaska Native, Native Hawaiian or Other Pacific Islander, and unknown races (missing or declined).

Per clinical protocol at TCH, all pediatric endocrinologists must document diabetes type in a designated section (“flowsheet”) of the EMR at each diabetes encounter, by selecting one of the options including T1D, T2D, steroid-induced diabetes, cystic fibrosis-related diabetes, gestational diabetes, MODY, neonatal diabetes, and unknown diabetes type. Per clinical protocol, patients with new-onset diabetes have islet autoantibodies, random C-peptide and random glucose tested at the time of diagnosis during hospital admission for new-onset diabetes teaching. This information is available for the treating pediatric endocrinologist at time of the first outpatient visit, along with other clinical and demographic characteristics. Patients not requiring hospital admission at the time of diagnosis due to milder presentation based on blood glucose and HbA1c are usually tested for missing autoantibody and/or C-peptide (with simultaneous glucose) at their outpatient assessment. Over 93% of patients with new onset diabetes have islet autoantibodies tested within the first 6 months of diagnosis. Using available clinical, biochemical, and immunological data, clinicians determine diabetes type and document accordingly at the first outpatient visit. If a patient’s presentation does not fit into one of the well-described diabetes types, clinicians select diabetes type as “unknown”. Diabetes type at first outpatient visit and at an outpatient visit that occurred between 3–4 years post-diagnosis were also extracted and were classified as one of the following: type 1, type 2, other (MODY steroid-induced, cystic fibrosis [CF]-related, other-not specified), and unknown.

### Statistical analyses

Patient characteristics were summarized by median with 25th and 75th percentile, mean with standard deviation, or frequency with proportion. Descriptive statistics were stratified by whether the type of diabetes was known or unknown at the first office visit after diabetes diagnosis, and reclassified or steady diabetes type 3 years post-diagnosis. The characteristics of the study population were compared using the Wilcoxon Rank Sum test or Pearson Chi-square test. Univariable and multivariable logistic regression models were used to identify baseline characteristics that were significantly associated with unknown or reclassified diabetes type. All the significant factors from univariable model were included in multiple logistic regression model. Multiple linear regression model was used to analyze if unknown or reclassified (based on baseline to 3–4 years post-diagnosis diabetes type, see Definitions below) diabetes type was significantly associated with HbA1c at 6–12 months after diagnosis after adjusting for age, gender, race, ethnicity, HbA1c at diagnosis, and duration from diabetes diagnosis to the HbA1c at 6–12 months. A significance level of 0.05 was used.

### Definitions

*Unknown* *diabetes* *type*: Diabetes of unknown type as documented by the treating pediatric endocrinologist in the EMR flowsheet section at each outpatient diabetes encounter.

*Reclassified* *diabetes* *type*: Diabetes type that was different at the time of the first outpatient visit and an outpatient visit between 3–4 years after diagnosis, both as documented by the treating pediatric endocrinologist in the EMR flowsheet. In the analysis, the diagnosis type at 3 years post-diagnosis was considered the final diagnosis type for the purpose of this study. Patients initially diagnosed with an unknown diabetes type who were then reclassified to a known diabetes type were not included in the count of reclassified diabetes type.

### Ethical approval

This study was approved and the requirement for informed consent was waived by the Baylor College of Medicine Institutional Review Board (IRB).

### Prior presentation

Parts of the content of this manuscript were presented at the American Diabetes Association’s 83rd Scientific Meeting in San Diego, CA, in June 2023.

## Results

We studied 2073 children with new onset diabetes who met our inclusion criteria (Entire Cohort). The median age [IQR] at diagnosis was 11.4 [6.2] years and 50% were male. Racial distribution was 75% White, 21% Black and 4% other races. Overall, 37% were Hispanic. The median HbA1c [IQR] at diagnosis was 11.4% [3.4] and median BMI [IQR] was 85 [39] percentile. Twenty-six percent presented with DKA at diagnosis. The most common diabetes types were T1D (73.3%) followed by T2D (21.2%) while diabetes type was unknown in 3% (n = 62) of the cohort at diagnosis of diabetes. The remaining 2.5% (n = 52) had “Other” types of diabetes (i.e., steroid-induced diabetes, cystic fibrosis-related diabetes, gestational diabetes, MODY or neonatal diabetes). Baseline characteristics are summarized in Tables 1 and 2.

**Table 1:** Demographics and clinical characteristics of patients with “unknown” diabetes type vs. known diabetes type, entire cohort (n = 2073).

Demographic or clinical characteristic	N	Overall cohort	Known diabetes type (n = 2011)	Unknown diabetes type (n = 62)	p-value
Age, years	2073	11.4 (6.2)	11.3 (6.2)	13.6 (3.5)	**< 0.001**
Male, n (%)	2073	1028 (50%)	1002 (50%)	26 (42%)	0.22
Race, n (%)	2023				0.34
White		1516 (75%)	1475 (75%)	41 (67%)	
Black or African American		417 (21%)	400 (20%)	17 (28%)	
Other		90 (4%)	87 (4%)	3 (5%)	
Ethnicity, n (%)	2042				0.06
Hispanic or Latino		756 (37%)	726 (37%)	30 (48%)	
Not Hispanic or Latino		1286 (63%)	1254 (63%)	32 (52%)	
BMI percentile at diagnosis	1562	85 (39)	84 (40)	97 (9)	**< 0.001**
GADA positive, n (%)	2073	1606 (77%)	1571 (78%)	35 (56%)	**< 0.001**
IA-2A positive, n (%)	2073	1559 (75%)	1525 (76%)	34 (55%)	**< 0.001**
Insulin autoantibody positive, n (%)	2073	1129 (54%)	1097 (55%)	32 (52%)	0.65
C-peptide at diagnosis, ng/mL	1407	0.54 (0.79)	0.55 (0.77)	1.36 (1.10)	**< 0.001**
Glucose at diagnosis, mg/dL	1188	312 (216)	313 (216)	280 (170)	0.56
HbA1c at diagnosis, %	1519	11.4 (3.4)	11.4 (3.4)	12.0 (3.5)	0.26
Presence of DKA at diagnosis, n (%)	2073	548 (26%)	544 (27%)	4 (6%)	**< 0.001**
Diabetes type at initial diagnosis, n (%)	2073				**< 0.001**
Type 1		1519 (73.3%)	1519 (75.5%)	0 (0%)	
Type 2		440 (21.2%)	440 (21.9%)	0 (0%)	
Other		52 (2.5%)	52 (2.6%)	0 (0%)	
Unknown		62 (3%)	0 (0%)	62 (100%)	

*DKA* diabetic ketoacidosis, *GADA* glutamic acid decaborxylase-65 antibody, *IA-2A* islet antigen 2 antibody.

Significant values are in bold.

**Table 2:** Demographics and clinical characteristics of patients with “unknown” diabetes type vs. known diabetes type, longitudinal cohort only (n = 1019).

Demographic or clinical characteristic	N	Longitudinal cohort	Known diabetes type (n = 992)	Unknown diabetes type (n = 27)	p-value
Diabetes type 3 years post-diagnosis, n (%)	1019				**< 0.001**
Type 1		831 (81.6%)	820 (82.7%)	11 (40.7%)	
Type 2		163 (16%)	154 (15.5%)	9 (33.3%)	
Other		18 (1.8%)	15 (1.5%)	3 (11.1%)	
Unknown		7 (0.7%)	3 (0.3%)	4 (14.8%)	

DKA: diabetic ketoacidosis, GADA: glutamic acid decaborxylase-65 antibody, IA-2A: islet antigen 2 antibody.

Unless otherwise specified, all continuous variables were expressed as median (IQR). Wilcoxon rank sum test and Chi-square test were used as appropriate. N is the number of non-missing values.

Significant values are in bold.

Compared to those with known diabetes type at diagnosis, children with unknown diabetes type were older (p < 0.001), less likely to be positive for GAD-65 or IA-2 autoantibodies (both p < 0.001), and had higher median BMI percentile (p < 0.001) and random C-peptide level (p < 0.001) (Table 1). They also had significantly lower percentage of DKA at diagnosis (p < 0.001). In a multivariable logistic regression analysis including age, sex, race, ethnicity, BMI percentile, C-peptide, presence of DKA, GADA positivity and IA-2A positivity, unknown diabetes type at diagnosis was associated with older age (p = 0.047), negative IA-2A (p = 0.005), lower C-peptide (p = 0.004), and absence of DKA (p = 0.024) (Table 3). After excluding patients with positive autoantibody status in a sensitivity analysis, older age, lower C-peptide and absence of DKA were still significantly associated with unknown diabetes type (all p < 0.05). In addition, African American race, Hispanic ethnicity, and higher BMI percentile emerged as additional significant factors in this model (all p < 0.05).

**Table 3:** Association of baseline characteristics with unknown diabetes types in a multivariable logistic regression analysis including age, sex, race, ethnicity, BMI percentile, C-peptide, presence of DKA, GADA positivity and IA-2A positivity.

Variable	OR	95% CI	p-value
Age	1.15	1.01–1.32	**0.047**
Sex	1.04	0.46–2.36	0.929
Black or African American Race	2.44	0.68–9.52	0.180
Other race	2.50	0.12–17.75	0.426
Not Hispanic or Latino Ethnicity	0.41	0.11–1.38	0.163
BMI percentile	1.01	0.99–1.04	0.260
GADA positivity	0.34	0.10–1.05	0.066
IA-2A positivity	0.17	0.04–0.56	**0.005**
C-peptide	0.54	0.35–0.78	**0.004**
Presence of DKA	0.23	0.05–0.72	**0.024**

*DKA* diabetic ketoacidosis, *GADA* glutamic acid decaborxylase-65 antibody, *IA-2A* islet antigen 2 antibody.

Significant values are in bold.

Of 2073 children, 1019 (49%) of them had follow up data available between 3 and 4 years after diabetes diagnosis (longitudinal subcohort) (Table 4). Individuals with longitudinal follow-up data were younger, more likely to be non-Hispanic, had lower BMI percentile and lower C-peptide at diagnosis compared to those without follow up data (Supplemental Table 1). There were no differences in sex, racial distribution, individual islet antibody positivity (GADA, IA–2A or IAA), glucose, HbA1c and presence of diabetic ketoacidosis at diagnosis. It is plausible that individuals with T1D phenotype are more likely to have longitudinal data and thus, are driving the differences between the groups as anecdotally follow-up rates are relatively lower in individuals with T2D.

**Table 4:** Demographics and clinical characteristics of patients with “reclassified” diabetes type from diagnosis to 3 years post-diagnosis vs. steady diabetes type.

Demographic or clinical characteristic	N	Overall cohort	Steady diabetes type (n = 984)	Reclassified diabetes type (n = 35)	p-value
Age, years	1019	10.7 (5.7)	10.5 (5.9)	13.6 (3.6)	**< 0.001**
Male, n (%)	1019	515 (51%)	496 (50%)	19 (54%)	0.65
Race, n (%)	1002				0.16
White		772 (77%)	748 (77%)	24 (71%)	
Black or African American		190 (19%)	180 (19%)	10 (29%)	
Other		40 (4%)	40 (4%)	0 (0%)	
Ethnicity, n (%)	1002				**< 0.001**
Hispanic or Latino		337 (34%)	314 (32%)	23 (66%)	
Not Hispanic or Latino		665 (66%)	653 (68%)	12 (34%)	
BMI percentile at diagnosis	670	82.7 (41.5)	81.0 (41.3)	98.2 (3.9)	**< 0.001**
GAD autoantibody positive, n (%)	1019	807 (79%)	790 (80%)	17 (49%)	**< 0.001**
IA-2 antibody positive, n (%)	1019	788 (77%)	773 (79%)	15 (43%)	**< 0.001**
Insulin autoantibody positive, n (%)	1019	565 (55%)	550 (56%)	15 (43%)	0.13
C-peptide at diagnosis, ng/mL	719	0.47 (0.66)	0.46 (0.60)	1.79 (1.05)	**< 0.001**
Glucose at diagnosis, mg/dL	608	316 (226)	321 (226)	246 (169)	**0.01**
HbA1c at diagnosis, %	747	11.4 (3.3)	11.5 (3.3)	10.3 (4.4)	**0.01**
Presence of DKA at diagnosis, n (%)	1019	272 (27%)	269 (27%)	3 (9%)	**0.01**
Diabetes type at initial diagnosis, n (%)	1019				**< 0.001**
Type 1		831 (82%)	814 (83%)	17 (49%)	
Type 2		136 (13%)	131 (13%)	5 (14%)	
Other		25 (1%)	12 (1%)	13 (37%)	
Unknown		27 (3%)	27 (3%)	0 (0%)	
Diabetes type 3 years post-diagnosis, n (%)	1019				**< 0.001**
Type 1		831 (82%)	825 (84%)	6 (17%)	
Type 2		163 (16%)	140 (14%)	23 (66%)	
Other		18 (2%)	15 (3%)	3 (9%)	
Unknown		7 (1%)	4 (0%)	3 (9%)	

Unless otherwise specified, all continuous variables were expressed as median (IQR). Wilcoxon rank sum test and Chi-square test were used as appropriate. N is the number of non-missing values. *DKA* diabetic ketoacidosis, *GADA* glutamic acid decaborxylase-65 antibody, *IA-2A* islet antigen 2 antibody.

In the longitudinal subcohort, 35 patients (3.4%) were identified as having a reclassified diagnosis of diabetes type 3 years after onset while diabetes classification remained steady in 984 patients (96.6%). Of these 35 patients with a reclassified diabetes type, approximately half of them were initially diagnosed as having T1D (49%, n = 17), five (14%) were diagnosed with T2D, and 13 (37%) were diagnosed with other diabetes types (Table 4). After 3 years, most of the patients with reclassified diabetes type were diagnosed with T2D (66%, n = 23), six patients were diagnosed with T1D (17%), three (9%) patients with other diabetes types, and three patients (9%) with unknown diabetes type (Table 4). As indicated in Methods, children with unknown diabetes type at diagnosis were not included in the category of children with reclassified diabetes type even if their diabetes was reclassified to a known type at the 3 year follow-up visit.

Compared to those with steady diabetes type 3 years post-diagnosis, children with reclassified diabetes type were older (p < 0.001), more likely to be of Hispanic ethnicity (p < 0.001) and negative GADA (p < 0.001) and IA–2A (p < 0.001) , and had higher C-peptide (p < 0.001) but lower glucose (p = 0.01) and HbA1c (p = 0.01) levels at diagnosis (Table 4). They also had significantly lower percentage of DKA at diagnosis (p = 0.01). In a multivariable logistic regression analysis including age, sex, race, ethnicity, BMI percentile, C-peptide, presence of DKA, GAD antibody positivity, IA–2 antibody positivity, HbA1c, and glucose, there was no individual variable associated with reclassified diabetes type after adjustment for confounding factors (data not shown).

Neither unknown nor reclassified diabetes type was significantly associated with HbA1c at 6–12 months post-diagnosis after adjusting for age, sex, race, ethnicity, HbA1c at diagnosis, and duration from diabetes diagnosis to the HbA1c at 6–12 months in a multivariable linear regression model (both p > 0.05, data not shown).

## Discussion

The prompt and accurate diagnosis of diabetes type is an ongoing issue in the pediatric population. We found that, in a racially and ethnically diverse cohort, the type of diabetes is unknown at the time of diabetes diagnosis or reclassified 3 years later in 6.4% of youth (one in 15 children) with diabetes mellitus. Guidelines for patient education and clinical management (e.g., schedule for screening of complications and associated conditions) differs by diabetes type^19^. Therefore, an imprecise diagnosis would lead the clinician to implement inappropriate education and management, potentially placing the patient at increased risk for negative health outcomes. Additionally, not receiving a diagnosis of diabetes type or having this reclassified over time could harm the trust built between families and their diabetes care providers. Therefore, accurate and timely diagnosis of diabetes type at the time of diabetes onset is crucial.

As expected, T1D was the most common diabetes type in our cohort, both at diagnosis and 3 years later. However, other diabetes types were more common 3 years after onset among children who at onset received an inaccurate diagnosis of diabetes type, that is, who were classified as having unknown type of diabetes or whose diabetes type was reclassified. This finding suggests a delay in diagnosing pediatric diabetes types other than T1D, such as T2D or MODY. At onset, children were over diagnosed as having T1D while other diabetes types were underdiagnosed. Hispanic ethnicity was significantly more frequent among the children with reclassified diabetes type (66%) compared with those with steady classification (33%), possibly due to the difficulties to accurately diagnose pediatric T2D at onset and the increasing rates of T2D disproportionately affecting Hispanic and Black youth^1^.

The misdiagnosis of T1D in a patient with T2D could result in unnecessary insulin treatment when diet and lifestyle modifications along with non-insulin medications may be more appropriate^20^. Insulin treatment increases the risk of hypoglycemia^21^, which is associated with adverse effects on the central nervous system and higher risk of cardiovascular events and mortality, especially during a severe episode^22^. In addition, insulin treatment is associated with significant emotional distress in individuals with diabetes and their families^23,24^. On the contrary, initial misclassification of T1D as another type of diabetes or lack of a timely diagnosis of T1D (i.e., unknown diabetes type) may place children at greater risk of DKA, a life-threatening complication of diabetes^25–27^. DKA is associated with several short- and long-term adverse health outcomes such as decline in memory and intelligence quotient^28^, altered brain structure^29^, kidney injury^30^, cerebral venous thrombosis^31^, decreased likelihood of having partial remission^32^ and negative impact on glycemic control over time^33,34^. Therefore, establishing an accurate diagnosis of diabetes type in a timely manner is of paramount importance for optimal health outcomes.

In the present study, older age, negative IA–2A, lower C-peptide, and absence of DKA at the onset of diabetes emerged as significant factors associated with unknown diabetes type limiting clinicians’ ability to determine diabetes type at the time of diagnosis of diabetes. Considering the biochemical and clinical characteristics of T1D and T2D^3,35^, this observation suggests that a lack of a typical characteristics of T1D at diagnosis of diabetes may lead to confusion in determination of diabetes type by clinicians. Furthermore, C-peptide levels measured at diagnosis are difficult to interpret. For example, although type 2 diabetes is typically associated with relatively elevated C-peptide levels, extreme hyperglycemia, ketosis and DKA (which can be observed at diagnosis of type 2 diabetes) cause a transient suppression of beta-cell function and thus, C-peptide levels in the range observed in type 1 diabetes. A substantial percentage of those with “unknown diabetes type” at the onset of diabetes were diagnosed with T2D at 3–4 years post-diagnosis. This aligns with our observation that a lack of T1D-suggestive phenotypic features in children plays an important role in delayed determination of diabetes type and underscores the urgent need for better diagnostic tools and biomarkers to confirm the timely diagnosis of T2D in children. Genetic risk scores alone (T1D and T2D genetic risk scores), or in combination with other metabolic and immunological markers may be useful as such tools. Dramatic increase in the incidence of pediatric T2D in recent decades^1^, more rapid beta-cell functional deterioration^36^ and earlier onset of diabetes complications^37–39^ in youth with T2D compared with their adult counterparts only augment the gravity of this public health problem and call all stakeholders to take immediate actions.

Delayed diagnosis or misdiagnosis of diabetes type at diagnosis of diabetes in children is due, in part, to limitations of the current classification system of diabetes^2^, which offers clinicians only limited options of diabetes types despite tremendous heterogeneity of diabetes^40^. Although distinct categorization of diabetes types seems clinically more practical in real-word settings, evidences supporting the complexity of diabetes and the existence of a spectrum of diabetes phenotypes with differential contributions of multiple pathophysiologic processes in concert with each other as suggested by Palette Model are accumulating^3,41^. This led to multiple efforts by us and other investigators looking into improved classification of diabetes types and subtypes to allow for personalized treatment approaches^5,10,16,42,43^. In one such effort, our group is currently working on to develop and validate improved models to differentiate between T1D and T2D in children using three existing T1D genetic risk scores in combination with islet antibodies and other available data at the time of diabetes onset in an NIH-funded study (i.e., The Diabetes Study in Children of Diverse Ethnicity and Race [DISCOVER])^44^.

The limitations of our study include its retrospective design and having 3 year follow-up data for only half of the cohort. In addition, our results may not be generalizable to patient populations without the information that we collect, including islet autoantibodies and C-peptide; in populations without this information, the prevalence of inaccurate diagnosis of diabetes type would likely increase. The study strengths include a large, racially/ethnically diverse pediatric patient population and availability of widely used biological and clinical data that allowed us to examine clinically impactful factors in the context of our research question.

In conclusion, one in 15 children is affected by inaccurate diagnosis of diabetes type in a racially and ethnically diverse pediatric diabetes population. Multifaceted effects of inaccurate diagnosis of diabetes type in health outcomes warrant further research to better understand the underlying root causes and offer impactful solutions. These efforts may include improved classification of diabetes and the incorporation in clinical practice of biomarkers (e.g., genetics) to characterize diabetes in children.

### Supplementary Information

Supplementary Table 1.

## Data Availability

The datasets generated during and/or analyzed during the current study are available from the corresponding author on reasonable request.
