# Supplementary material for: Inaccurate diagnosis of diabetes type in youth: prevalence, characteristics, and implications
Source: Sci Rep. 2024 Apr 17;14:8876. doi: 10.1038/s41598-024-58927-6 (PMC11024140; doi:10.1038/s41598-024-58927-6)
Supplement: Supplementary file 1 — Supplementary Table 1. [file 41598_2024_58927_MOESM1_ESM.docx]

**Supplemental Table 1.** Demographics and clinical characteristics of patients with a follow-up data (i.e., longitudinal cohort) vs. without follow-up data. (DKA: diabetic ketoacidosis, GADA: glutamic acid decaborxylase-65 antibody, IA-2A: islet antigen 2 antibody)

| **Demographic or clinical characteristic** | **N** | **Overall cohort** | **Longitudinal Cohort (n=1019)** | **Individuals without follow-up data (n=1054)** | **p-value** |
| --- | --- | --- | --- | --- | --- |
| **Age,** years | 2073 | 11.4 (6.2) | 10.7 (5.7) | 12.3 (6.5) | <0.001 |
| **Male**, n (%) | 2073 | 1028 (50%) | 515 (51%) | 513 (49%) | 0.4 |
| **Race**, n (%) | 2023 |  |  |  | 0.09 |
| White |  | 1516 (75%) | 772 (77%) | 744 (73%) |  |
| Black or African American |  | 417 (21%) | 190 (19%) | 227 (22%) |  |
| Other |  | 90 (4%) | 40 (4%) | 50 (5%) |  |
| **Ethnicity**, n (%) | 2042 |  |  |  | 0.002 |
| Hispanic or Latino |  | 756 (37%) | 337 (34%) | 419 (40%) |  |
| Not Hispanic or Latino |  | 1286 (63%) | 665 (66%) | 621 (60%) |  |
| **BMI percentile at diagnosis** | 1562 | 85 (39) | 83 (41) | 88 (36) | <0.001 |
| **GADA positive**, n (%) | 2073 | 1606 (77%) | 807 (79%) | 799 (76%) | 0.07 |
| **IA-2A positive**, n (%) | 2073 | 1559 (75%) | 788 (77%) | 771 (73%) | 0.03 |
| **Insulin autoantibody positive**, n (%) | 2073 | 1129 (54%) | 565 (55%) | 564 (54%) | 0.38 |
| **C-peptide at diagnosis**, ng/mL | 1407 | 0.54 (0.79) | 0.47 (0.66) | 0.61 (1) | <0.001 |
| **Glucose at diagnosis,** mg/dL | 1188 | 312 (216) | 316 (226) | 306 (209) | 0.57 |
| **HbA1c at diagnosis**, % | 1519 | 11.4 (3.4) | 11.4 (3.3) | 11.4 (3.5) | 0.17 |
| **Presence of DKA at diagnosis**, n (%) | 2073 | 548 (26%) | 272 (27%) | 276 (66%) | 0.79 |
